# Neodb: a comprehensive neoantigen database and discovery platform for cancer immunotherapy

**DOI:** 10.1093/database/baad041

**Published:** 2023-06-13

**Authors:** Tao Wu, Jing Chen, Kaixuan Diao, Guangshuai Wang, Jinyu Wang, Huizi Yao, Xue-Song Liu

**Affiliations:** School of Life Science and Technology, ShanghaiTech University, 393 Middle Huaxia Road, Pudong, Shanghai 201203, China; Shanghai Institute of Biochemistry and Cell Biology, Chinese Academy of Sciences, 320 Yue Yang Road, Shanghai 200031, China; University of Chinese Academy of Sciences, 1 Yanqihu East Rd, Huairou District, Beijing 101408, China; School of Life Science and Technology, ShanghaiTech University, 393 Middle Huaxia Road, Pudong, Shanghai 201203, China; School of Life Science and Technology, ShanghaiTech University, 393 Middle Huaxia Road, Pudong, Shanghai 201203, China; School of Life Science and Technology, ShanghaiTech University, 393 Middle Huaxia Road, Pudong, Shanghai 201203, China; School of Life Science and Technology, ShanghaiTech University, 393 Middle Huaxia Road, Pudong, Shanghai 201203, China; School of Life Science and Technology, ShanghaiTech University, 393 Middle Huaxia Road, Pudong, Shanghai 201203, China; School of Life Science and Technology, ShanghaiTech University, 393 Middle Huaxia Road, Pudong, Shanghai 201203, China; Shanghai Clinical Research and Trial Center, 1599 Keyuan Road, Pudong New Area, Shanghai 201203, China

## Abstract

Neoantigens derived from somatic deoxyribonucleic acid alterations are ideal cancer-specific targets. However, integrated platform for neoantigen discovery is urgently needed. Recently, many scattered experimental evidences suggest that some neoantigens are immunogenic, and comprehensive collection of these experimentally validated neoantigens is still lacking. Here, we have integrated the commonly used tools in the current neoantigen discovery process to form a comprehensive web-based analysis platform. To identify experimental evidences supporting the immunogenicity of neoantigens, we performed comprehensive literature search and constructed the database. The collection of public neoantigens was obtained by using comprehensive features to filter the potential neoantigens from recurrent driver mutations. Importantly, we constructed a graph neural network (GNN) model (Immuno-GNN) using an attention mechanism to consider the spatial interactions between human leukocyte antigen and antigenic peptides for neoantigen immunogenicity prediction. The new easy-to-use R/Shiny web–based neoantigen database and discovery platform, Neodb, contains currently the largest number of experimentally validated neoantigens. In addition to validated neoantigen, Neodb also includes three additional modules for facilitating neoantigen prediction and analysis, including ‘Tools’ module (comprehensive neoantigen prediction tools); ‘Driver-Neo’ module (collection of public neoantigens derived from recurrent mutations) and ‘Immuno-GNN’ module (a novel immunogenicity prediction tool based on a GNN). Immuno-GNN shows improved performance compared with known methods and also represents the first application of GNN model in neoantigen immunogenicity prediction. The construction of Neodb will facilitate the study of neoantigen immunogenicity and the clinical application of neoantigen-based cancer immunotherapy.

**Database URL** https://liuxslab.com/Neodb/

## Introduction

Somatic deoxyribonucleic acid (DNA) alterations are the major driving forces for the clonal evolution of cancer cells. Neoantigens derived from these somatic DNA alterations are ideal cancer-specific targets. Cancer neoantigen vaccines and neoantigen-targeting T cell receptor–engineered T cells have demonstrated clinical effects in treating cancer (1). However, accurate prediction of the immunogenicity encoded by the somatic DNA alteration is still a significant scientific challenge. Experimental evidence is the gold standard for determining whether neoantigens are immunogenic. Recently, many scattered experimental evidences suggest that some neoantigens are immunogenic, and comprehensive collection of these experimentally validated neoantigens is still lacking. Furthermore, public neoantigens derived from recurrent somatic DNA alterations can be targets for a significant portion of cancer patients compared with personalized neoantigens (2), and a systematic evaluation and providing the list of potential neoantigens derived from these recurrent DNA mutations is also an urgent need.

**Figure 1.**
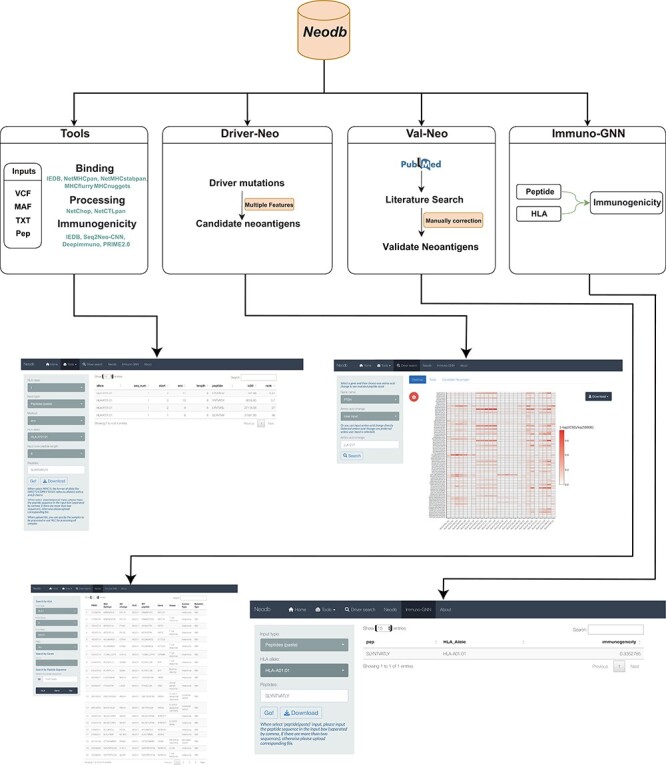
A comprehensive neoantigen database and discovery platform. An overview of the Neodb includes four modules: (i) the Tools module, which integrates the commonly used tools in the neoantigen prediction process; (ii) the Driver-Neo module, which includes the list of candidate neoantigens for common driver mutations predicted by combining multiple features; (iii) the Val-Neo module, which contains manually collected experimentally validated neoantigen peptide data; (iv) the Immuno-GNN model, which is a novel immunogenicity prediction tool.

In order to address the above-mentioned challenges, we developed the Neodb database. The database mainly consists of four modules: the integrated web server of comprehensive neoantigen prediction tools (‘Tools’ module); the manually curated experimentally validated immunogenic neoantigen dataset (‘Val-Neo’ module); a list of potential neoantigens derived from common driver mutations with multiple features (‘Driver-Neo’ module) and a novel immunogenicity prediction tool constructed based on a graph neural network (GNN) (‘Immuno-GNN’ module). Neodb has been developed with R version 4.1 and Shiny version 1.5. These data and tools can be retrieved, applied and visualized in a user-friendly manner (Figure 1).

## Materials and methods

### Data collection and curation

The data of our ‘Val-Neo’ module was curated manually from published immunology literatures and existing databases. Optimized keyword combinations were applied in the PubMed Advanced Search Builder tool, and we kept searched results in the limited time range between January 2000 and March 2022, resulting in 848 research articles. In order to ensure the reliability of the data, we conducted two rounds of literature checks (Supplementary Figure 1A and B). We first read the abstract of these papers to check whether the article might contain the neoantigens data we needed, and we further read the article body and supplementary materials to obtain the neoantigen peptide sequence and corresponding meta-information, including validation assay, human leukocyte antigen (HLA) restriction, mutation position, cancer type and wild-type (WT) sequence. Immunogenic neoantigens collected in our database were validated by the following assays: Cr-release assays, cytometric bead array, enzyme-linked immunosorbent assay, enzyme-linked immunospot (ELISpot), interferon-γ release assay, mutation-associated neoantigen functional expansion of specific T cells (3), multimer staining, T cell proliferation and T cell activation. After integrating data from other databases and quality control steps, we obtained 1463 validated neoantigen instances, with 1277 HLA-I and 186 HLA-II restrictions, respectively.

The common driver mutations were obtained from OncoVar database (4) which systematically prioritizes the oncogenic ability of somatic mutations and cancer genes. We filtered the driver mutations found in common cancer genes, resulting in 451 driver genes and 3068 driver mutations. The common HLA alleles of Chinese population were obtained from He et al. (5), which analyzed 812 211 unrelated volunteer donors from the China Marrow Donor Program, and we choose the HLA-I alleles labeled as ‘common’ and obtain 106 HLA-I alleles with a minimum frequency of 0.14%.

### Prediction of potential neoantigens from driver mutations

For each common driver mutation, we first used ANNOVAR (6) to obtain the mutant (MT) and WT sequences of the protein and then extracted the 8–14-mer MT and WT peptides surrounding the changed amino acid caused by the mutation. Then, we predicted the binding affinity (IC50) between these peptides and the common 106 HLA alleles using NetMHCpan-4.1 and filtered binders by a threshold of %Rank <2. The prediction results are displayed in the form of dynamic heatmaps in our ‘Driver-Neo’ module server, and users can visualize results using different thresholds. In order to obtain peptides that are more likely to become neoantigens, we applied additional filter conditions: differential agretopicity index (7) (DAI, defined as IC50 of WT divided by IC50 of MT peptide), which refers to the degree to which the binding capacity of a neoantigen differs from its WT counterpart (DAI > 1); the binding stability of protein-major histocompatibility complex (predicted by Netstabpan, %Rank < 2%); proteasomal C-terminal cleavage and transporter-associated with antigen processing (TAP) transport efficiency (predicted by NetCTLpan, combined %Rank < 2) and immunogenicity score predicted by multiple methods including DeepImmuno, Seq2neo-convolutional neural network (CNN), PRIME and Immuno-GNN (predicted positive in more than two methods; threshold used for DeepImmuno, Seq2neo-CNN and Immuno-GNN was 0.8, while for PRIME, the %Rank < 2 was used). We ended up with 20 971 candidate neoantigens that passed these screening criteria.

### Construction of the immunogenicity prediction model

The training data were obtained from Immune Epitope Database (IEDB). The filters are linear sequence, positive assays, negative assays, no B cell assays, no major histocompatibility complex (MHC) assays, MHC restriction type—Class I, host: *Homo sapiens* (human) and T cell assays—cytokine release, cytotoxicity. To ensure the high quality of our training data, we performed a series of data cleaning and preprocessing steps (Figure 4A): data entries without 4-digit HLA alleles and enough explicit experimental information were removed. For negative samples, the number of experiments performed should be >4 (that is, at least four experiments were all negative); for positive samples, the number of subjects responded should be at least one. Finally, all redundant peptide–HLA instances were discarded, leaving 8412 training data, including 3467 positive data and 4945 negative data. Then, the dataset is divided according to the sample type: 70% is used as the training set and 30% is used as the validation set (that is, the training set contains 70% positive and negative samples) for hyperparameter tuning. After hyperparameter tuning, we trained the model on whole dataset in a 10-fold cross-validation manner. We regard each amino acid of peptide and HLA (only keep 34 length pseudo-sequence used by NetMHCpan) as a node of the graph, and the feature encoding method of the node is as follows: first, the 566 features related to the physical and chemical properties of amino acids are obtained from the AAindex1 database, and the missing values are removed, remaining 553 features. These features were robustly normalized and then subjected to principal component analysis, retaining the top 20 principal components as features of amino acid nodes. Since we do not know how the nodes are connected, we construct the fully connected graph, where each two nodes has an edge. The GNN model we use is a TransformerConv with an attention mechanism that learns weights between different nodes and adds the GraphNorm for normalization of node features across the graph (Figure 4B). In order to obtain the information of the entire graph, we add a virtual node (this node is connected to all other nodes), and after the message passing of the 3-layer TransformerConv, we can get the embedding of this virtual node. This embedding vector is fed into a layer of multilayer perceptron to obtain the final immunogenicity prediction value. The loss function we used is binary cross entropy loss. We use grid search for hyperparameter tuning, and the optimal parameters obtained are shown in Supplementary Table S1. The positive samples of the independent test data were from the Val-Neo module of Neodb database we constructed. Since the supported prediction length of most methods is 9–10, we kept the data of these peptide lengths for testing, and the negative samples were obtained from the TESLA datasets, and the data that appeared in the training set were removed, resulting in 1220 test data entries with 736 positive and 484 negative items. We used these data to compare the performance of four other methods (Seq2neo-CNN, DeepImmuno, IEDB and PRIME2.0), and since these methods support different peptide lengths and HLA types, the size of the test data is also different (Figure 4D). The metrics we used to compare performance include sensitivity, F1 score and TopK metric (the proportion of positive samples in the top K samples with the highest predicted value).

### Implement

Neodb has been developed with R version 4.1 and Shiny version 1.5. The GNN model was implemented by Python version 3.8, PyTorch version 1.11.0 and torch-geometric version 2.0.4. The model was trained on A5000 GPU.

## Results

### Integrated tool for neoantigen discovery

To be recognized by T cells, the neoantigen peptides in tumor cells must undergo antigen presentation and T cell recognition processes (8), including proteasome cleavage, transport into the endoplasmic reticulum by TAP, bind to HLA and then be presented to the cell surface to activate T cells (Figure 4A). There are currently a series of machine learning and deep learning–based tools to predict these processes. In Neodb ‘Tools’ module, we divided these tools into three categories: antigen processing, antigen binding and immunogenicity prediction, integrated these tools into a unified interface and build an easy-to-use web server. For antigen processing, it includes Netchop (9) and NetCTLpan (10) which can predict proteasome cleavage and TAP transport efficiency, respectively; antigen binding includes IEDB tools, which contains 10 different tools, NetMHCpan (11), MHCflurry (12) and MHCnugget (13); immunogenicity prediction includes IEDB tool, Seq2Neo-CNN (14), DeepImmuno (15) and PRIME2.0 (16). Notably, our tools module supports the input of common variant file format, including MAF, VCF and TXT, as well as batch peptide input (PEP file). The example file can be downloaded from About page.

### Database of validated neoantigens

**Figure 2.**
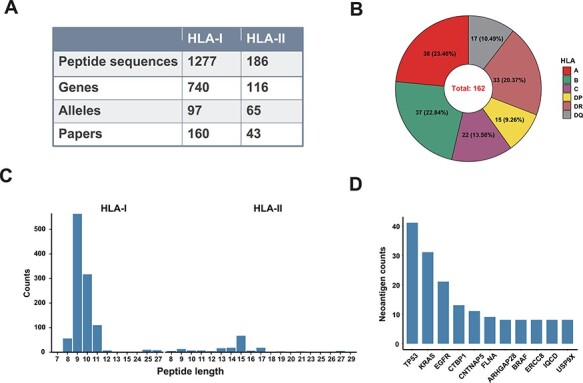
A data summary for Val-Neo. (**A**) A comparison of counts of neoantigens, genes, alleles and research papers between HLA-I and HLA-II. (**B**) Distribution of different HLA alleles. (**C**) Distribution of peptide lengths of different HLA types. (**D**) Top 10 genes with the most number of validated neoantigens.

**Figure 3.**
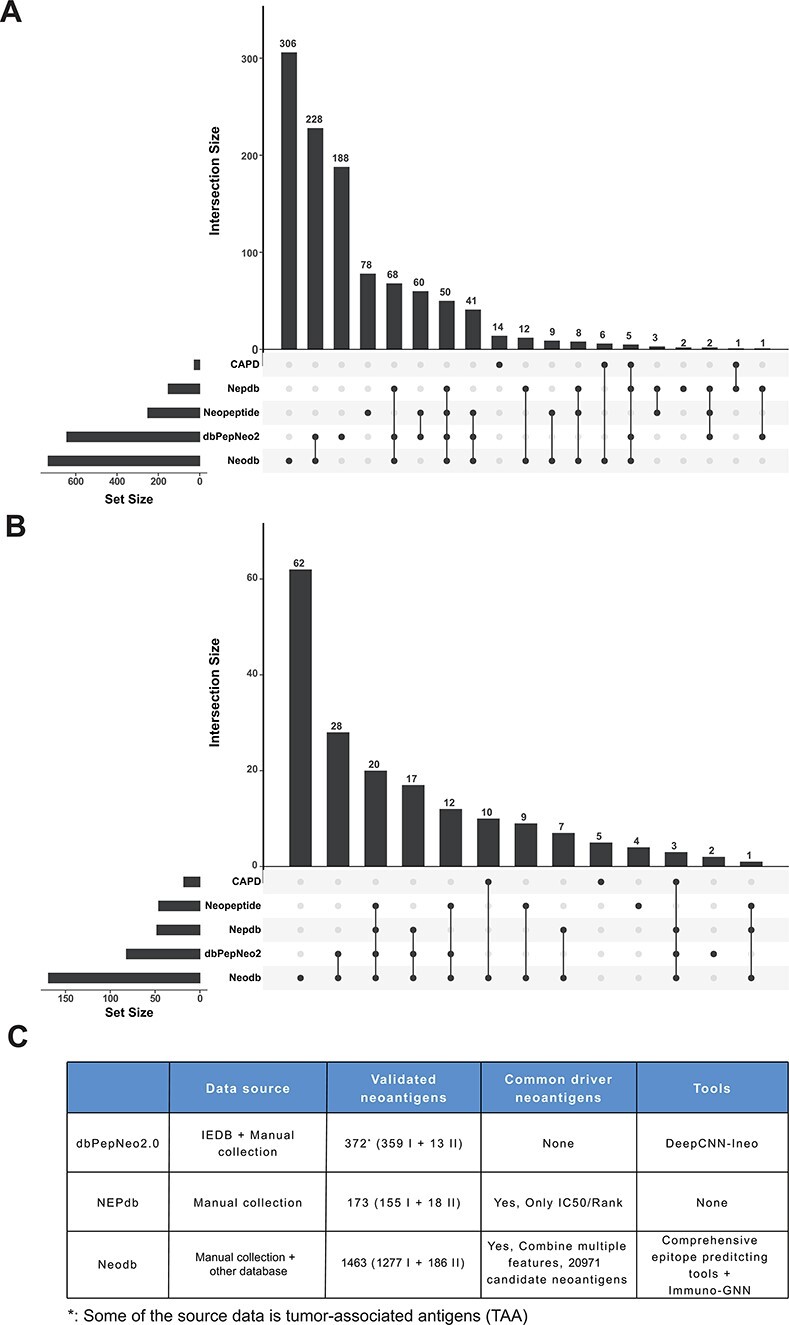
Comparison with other databases. (**A**) The amount of neoantigen–HLA pairs of different database and their overlap. (**B**) The amount of research papers of different databases and their overlap. (**C**) Comparison of different databases, including data sources, the number of validated neoantigens and whether there is a list of candidate neoantigens derived from common cancer driver mutations. The neopeptide and cancer antigenic peptide database (CAPD) only have the neoantigen peptide sequence and do not include tools and other resources, therefore they are not shown in the table.

‘Val-Neo’ module is a collection of experimentally validated immunogenic neoantigens. We used the optimized keywords to extensively search relevant literatures (Supplementary Figure S1). Currently, our database contains 1463 neoantigenic peptides from 206 papers (Figure 2A). Compare to other databases, including Nepdb (17) and dbPepNeo2.0 (18), our database represent the most comprehensive validated neoantigen database and has the largest number of neoantigens and papers that do not overlap with other databases (Figure 3A–C). Although there are some other databases containing T cell epitope data, such as SYFPEITHI and IEDB, most of the data contained in them are not related to cancer or are only tumor-associated antigens rather than neoantigens, and data of some databases are not readily accessible (such as SYFPEITHI). The neoantigens in our database are either validated *in vivo* by clinical vaccine immunizing or by *in vitro* immunological experiments, which can activate specific T cell responses. Meta-information for each neoantigens includes HLA type (I or II), amino acid changes, gene name, validation experiment, tested immune cell type, cancer type and source literature ID. The distribution of neoantigen peptide length, HLA alleles and the top 10 genes with the largest number of neoantigens is shown in Figure 2B–D. The sequence logos of different peptide lengths in ‘Val-Neo’ database are shown in Supplementary Figure S2. It can be seen that the amino acids at the second and last positions have the highest conservation, indicating that these two sites may be the anchor positions for the binding of peptides. Users can conveniently search on ‘Val-Neo’ database interface based on the meta-information of neoantigens. In addition, users can also input specific sequences, and the most similar sequences in the database and their similarity scores by sequence alignment will output.

### Candidate neoantigens for common cancer driver mutations

**Figure 4.**
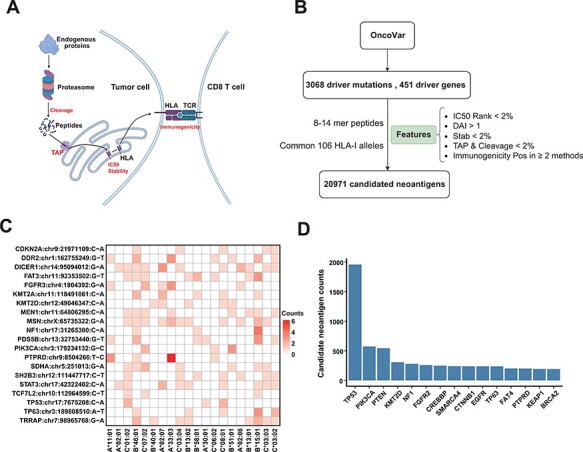
Candidate neoantigens of common cancer driver mutations. (**A**) The process of antigen presentation, including proteasome cleavage, transport into the endoplasmic reticulum by TAP, binding to HLA and then being presented to the cell surface to activate T cells. The processes of cleavage, translocating into endoplasmic reticulum by TAP, binding to HLA molecule and interacting with TCR are key steps in the antigen presentation process, so we use these features to filter neoantigens produced by common driver mutations as shown in (**B**) illustration of procedures for obtaining the list of potential neoantigens from common driver mutations by multiple features. (**C**) Common driver mutations that generate the top 20 amount of neoantigens and corresponding HLA allele (only show HLA alleles with top 20 population frequency). (**D**) The top 15 genes that can generate the largest number of potential neoantigens.

To provide a reliable list for ‘Off-the-Shelf’ neoantigens derived from common driver mutations, we used comprehensive features to filter the potential neoantigens from recurrent driver mutations. We obtained 3068 recurrent driver mutations from 451 genes from the OncoVar database (Materials and Methods section). For each non-synonymous mutation, we extracted the 8–14-mer MT and WT peptides surrounding the changed amino acid caused by the mutation. We then used the current state-of-the-art peptide–HLA binding prediction algorithms (NetMHCpan-4.1) to predict the affinity of these peptides to 106 HLA alleles that are frequent in Chinese population (see Supplementary Figure S3 for the distribution of HLA alleles). In order to obtain peptides that are more likely to become neoantigens, we employed multiple features (Figure 4A) for neoantigen filtering (19), including proteasomal cleavage, TAP transport efficiency, DAI, peptide–HLA binding stability and probability of activating T cells (Figure 4B; Materials and Methods section). After such filtering procedures, we got a list of 20 971 candidate neoantigens. Common driver mutations that generate the top 20 amount of neoantigens and corresponding HLA allele are shown in Figure 4C. Figure 4D shows the top 15 genes that can generate the largest number of potential neoantigens. The prediction results are displayed in the form of dynamic heatmaps in ‘Driver-Neo’ module server, and users can visualize results using different thresholds. Users can also retrieve driver mutations and potential neoantigens list for every gene.

### GNN model for immunogenicity prediction

**Figure 5.**
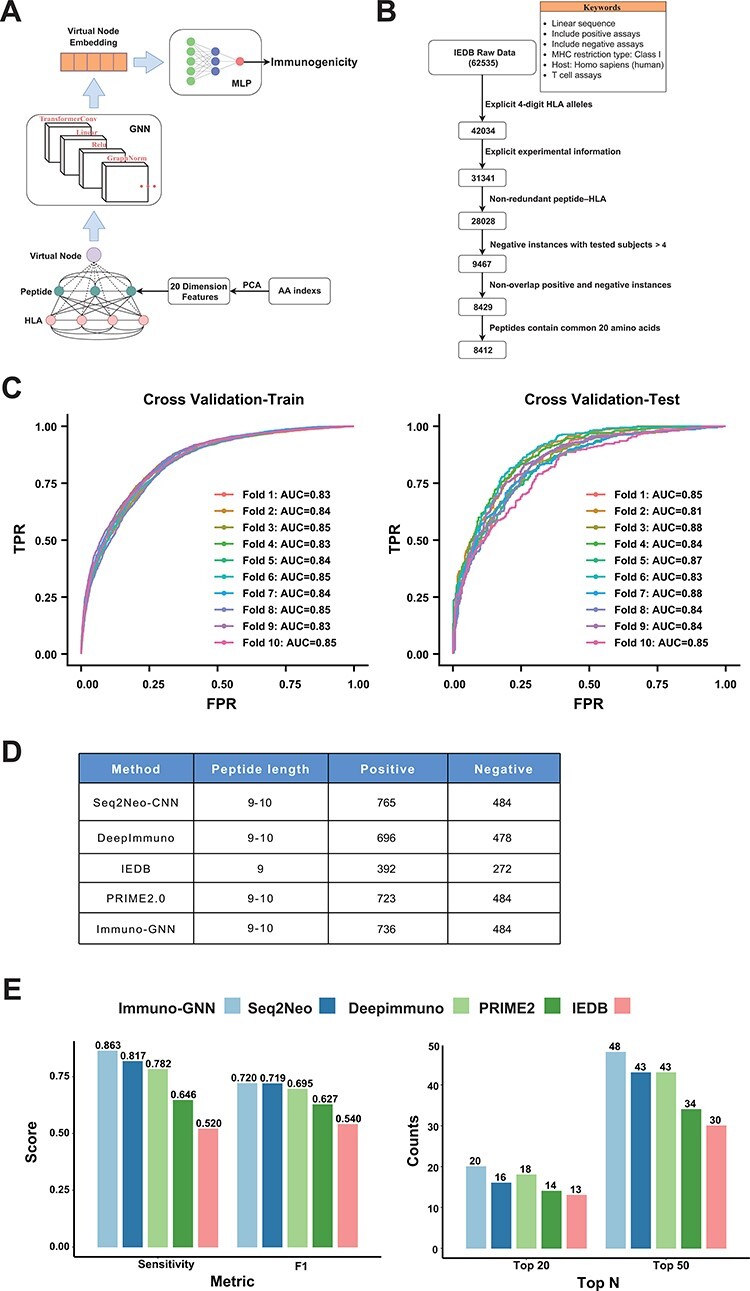
GNN model for immunogenicity prediction. (**A**) Architecture of the model for predicting immunogenicity based on GNNs (Immuno-GNN). (**B**) The step-by-step training data filter process, (**C**) ROC curve of Immuno-GNN on 10-fold cross-validation of the filtered IEDB training dataset. ROC, receiver operating characteristic; AUC, area under curve; TPR, true-positive rate; FPR, false-positive rate. (**D**) Validation data summary of different immunogenicity prediction tools, these data were extracted from our Neodb database. (**E**) Comparison of multiple immunogenicity prediction tools based on our collection of validated neoantigen data.

As one demonstration of the application of Neodb database, we construct a novel GNN-based immunogenicity prediction model. As a new deep learning architecture that has recently emerged, GNN has unique advantages in dealing with irregular data structures (20). Currently, fully connected neural networks and CNNs have been used in immunogenicity prediction, and these known methods treat peptides and HLA sequences as linear structures, ignoring their interactions, while GNN with attention mechanism can capture the interactions between different amino acids of HLA and antigen peptides. We regard each amino acid of a peptide and HLA as a node and construct the fully connected graph (each two nodes has an edge). The GNN architecture (Immuno-GNN) we constructed is shown in Figure 5A (details in Materials and Methods section). For the training of the model, we analyzed validated immunogenicity molecular assays from the IEDB (19 June 2022). Through multi-step data cleaning and filtering (Figure 5B; Materials and Methods section), we obtained 8412 training data (including 3467 positive samples and 4945 negative samples) and then divided the dataset into train and validation sets by the ratio of 7:3 for hyperparameter tuning (model hyperparameters are shown in Supplementary Table S1; training dataset are shown in Supplementary Table 2). Then, we trained the model on whole dataset in a 10-fold cross-validation manner (Figure 5C). We validated the model using the immunogenic neoantigen data collected in the ‘Val-Neo’ module of Neodb database (Materials and Methods section; Figure 5D; validation dataset is shown in Supplementary Table S3) and found that our model outperforms existing models on the sensitivity, F1 score and TopK metric (the proportion of positive samples in the top K samples with the highest predicted value, Figure 5E). The ‘Immuno-GNN’ module was built based on this immunogenicity prediction model, and users can easily get prediction results from their customized peptide–HLA input.

### Web interface

In Neodb, users can use different search or analysis modules from the home page or tabs at the top (Figure 1). In the Tools module and Immuno-GNN module, users can provide peptide sequences or mutation files (such as VCF and MAF file formats); in the Tools module, the parameters that users can set include HLA type, input file type, prediction method, HLA allele and predicted peptide lengths, the prediction results are displayed in a tabular form and the user can download the result file (csv format) after the run completed. In the Val-Neo and Driver-Neo modules, users can search for the neoantigen peptide sequences that we have collected or predicted. In the Driver-Neo module, users can search for corresponding neoantigens from driver mutations based on gene names and amino acid changes. The results are displayed in heatmaps and tables, and the list of candidate driver mutations filtered by various neoantigen characteristics is also output. In the Neodb model, users can search experimentally verified neoantigens according to HLA type, HLA allele, PubMed Identifier (PMID) and gene name. In addition, we also provide a sequence similarity search, outputting the most similar neoantigen in the database according to the sequence provided by the user. The results display the meta-information of the neoantigen in tabular form, including sequence, experiment type, PMID, gene name and corresponding HLA allele. Users can download example files (VCF, MAF and PEP formats) and all experimental verification neoantigen data in the About page. The About also provides the tutorial for our web services.

## Conclusion and discussion

In conclusion, we have developed a comprehensive and easy-to-use neoantigen discovery platform, including the manual collection of the largest experimentally validated neoantigens peptide database to date (including 1463 validated neoantigen instances with 1277 HLA-I and 186 HLA-II restriction, respectively), a list of 20 971 candidate neoantigens from common driver mutations and integration of a series of neoantigen prediction tools (including the novel Immuno-GNN tool developed here). Although our database contains most of the currently known experimentally validated neoantigens, it may not be exhaustive and there may be other potentially immunogenic neoantigens that have not been discovered or validated. Therefore, our database needs to be continuously updated to provide more abundant resources. The Immuno-GNN developed here is a novel immunogenicity prediction tool based on GNN, and further optimization is required to capture complex interactions between HLA and neoantigenic peptides. In addition, immunogenicity prediction results also need further experimental verification. We hope that Neodb will become a valuable resource to facilitate the study of neoantigen immunogenicity and the application of neoantigen-based immunotherapy.

## Supplementary Material

baad041_Supp

## Data Availability

All data and resources of Neodb are freely available at https://liuxslab.com/Neodb/ and https://github.com/XSLiuLab/Neodb.
