# Supplementary material for: Neodb: a comprehensive neoantigen database and discovery platform for cancer immunotherapy
Source: Database (Oxford). 2023 Jun 13;2023:baad041. doi: 10.1093/database/baad041 (PMC10263465; doi:10.1093/database/baad041)

**Supp Fig.1 Manually search experimentally-validated neoantigens from published literatures.**

A

B

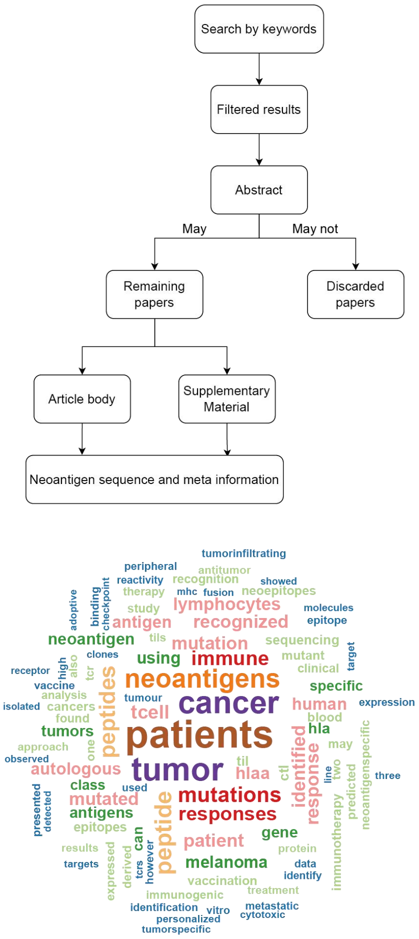


**(A)** Literature search and data collection procedures **(B)** Word cloud drawn from the abstracts of final 203 literatures which contain experimentally validated neoantigens.

## Supp Fig.2 The sequence logo of different length validated neoantigens.

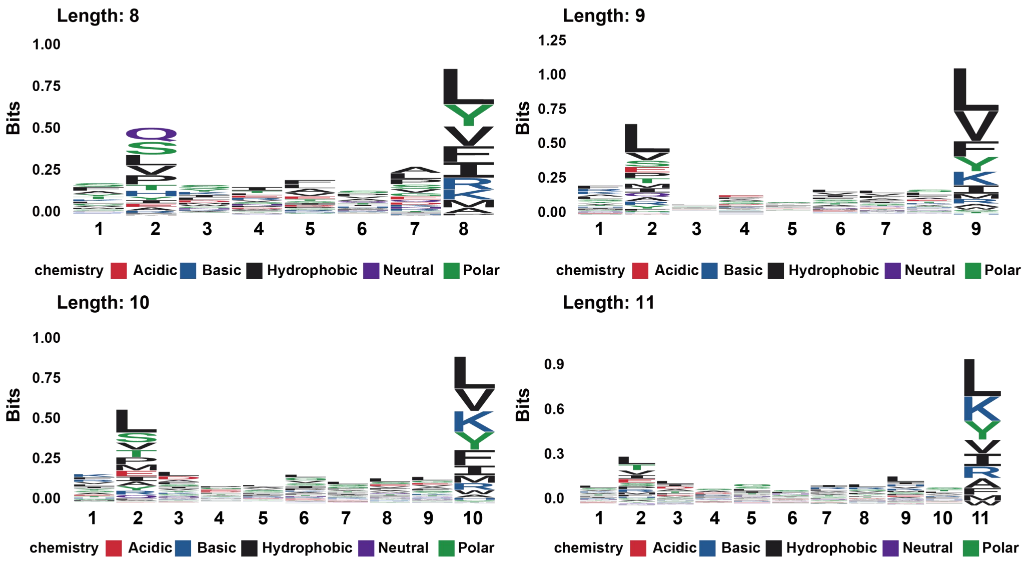

**Supp Fig.3 The distribution of HLA allele frequency in the Chinese population, and the top 20 most frequent HLA alleles of each sub-type were selected.**

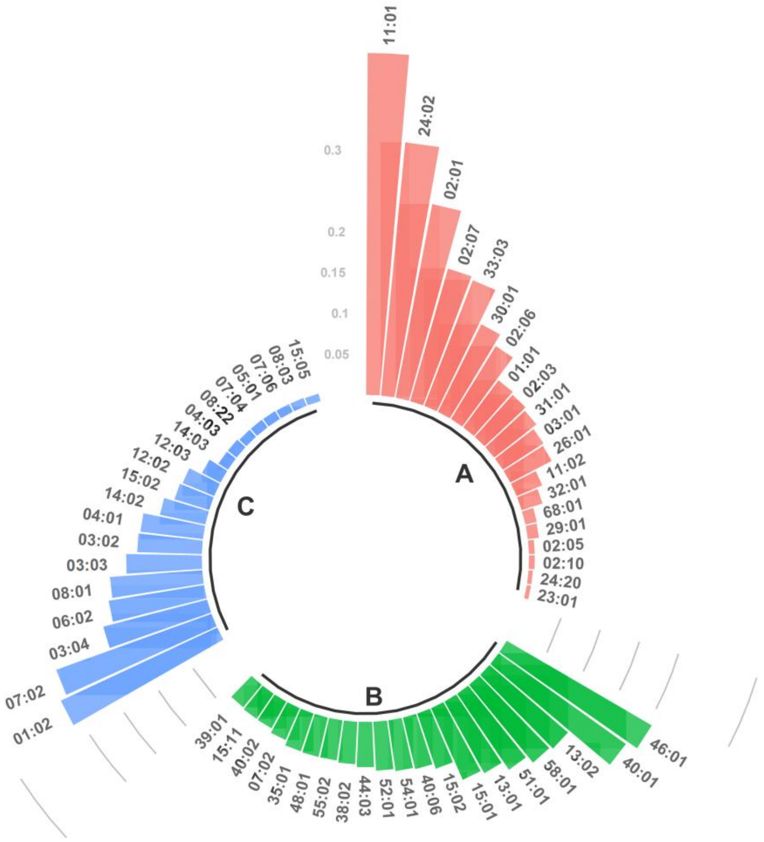

**Supp Fig.4 Heatmap of attention weight between HLA pseudo-sequence (34 amino acids length) and peptide sequence (9 amino acids length) position. The number on the right of each row indicates the sum of the weights of all HLA position in this row.**

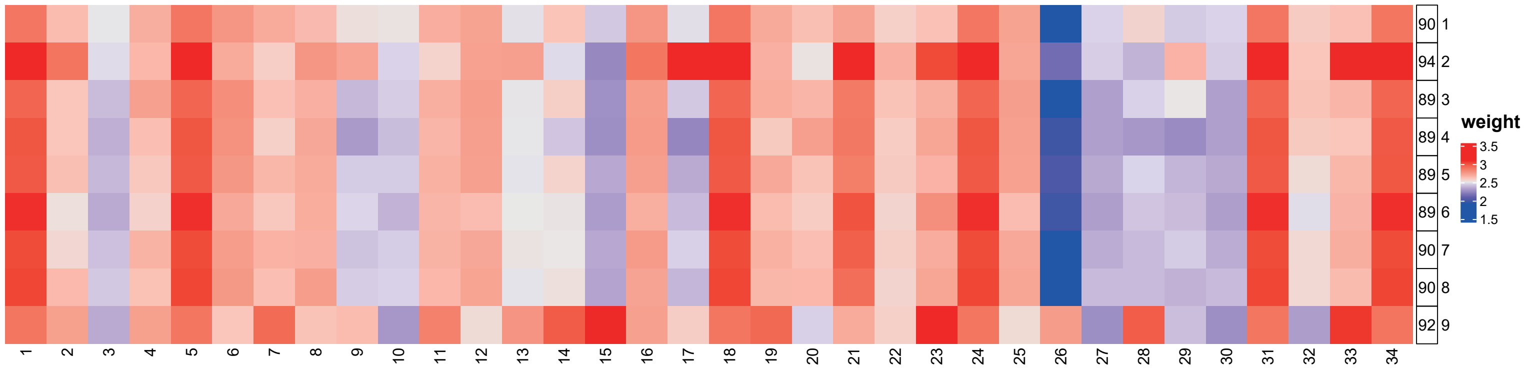

Supplement: baad041_Supp [file baad041_supp.zip › suppl_data/Neodb_Database_supp.pdf]
